# Emerging Neurovascular Interventions for the Prevention of Cerebral Vasospasm and Delayed Cerebral Ischemia After Aneurysmal Subarachnoid Hemorrhage: A Systematic Review

**DOI:** 10.7759/cureus.111891

**Published:** 2026-07-01

**Authors:** Dushan Bosotov, Mukhtar Ali Syed, Faiq Aftab, Faraz Sohail, Ravleen Chowdhary, Hamza Yamout, Muhammad Junaid, Alishba Khan, Geetanjali Khot, Syed Ali A Rahat, Ibrahim R Raza

**Affiliations:** 1 Neurology, American University of Antigua College of Medicine, Coolidge, USA; 2 Internal Medicine, Government Medical College and Hospital, Chhatrapati Sambhajinagar, IND; 3 Internal Medicine, Urgench State Medical Institute, Urgench, UZB; 4 Neurology, University of Health Sciences, Lahore, PAK; 5 Intrenal Medicine, Hamdard Institute of Medical Sciences and Research, New Delhi, IND; 6 Internal Medicine, Beirut Arab University, Beirut, LBN; 7 Accident and Emergency Medicine, Bahawal Victoria Hospital, Bahawalpur, PAK; 8 Internal Medicine, Liaquat National Hospital and Medical College, Karachi, PAK; 9 Orthopedics and Trauma, Navalaksha Fracture and Accidental Hospital, Sangli, IND; 10 General Surgery, Osh State University, Osh, KGZ; 11 Internal Medicine, Allama Iqbal Medical College, Lahore, PAK

**Keywords:** aneurysmal subarachnoid hemorrhage, cerebral vasospasm, cilostazol, clazosentan, delayed cerebral ischemia, endovascular therapy, intra arterial nimodipine, neuromodulation, neurovascular intervention, nicardipine

## Abstract

Aneurysmal subarachnoid hemorrhage remains a major cause of neurological morbidity and mortality, with delayed cerebral ischemia and cerebral vasospasm representing important contributors to secondary brain injury and unfavorable functional recovery. Although conventional management strategies such as nimodipine administration, hemodynamic augmentation, and endovascular rescue therapy remain central to clinical practice, growing evidence suggests that delayed cerebral ischemia is a multifactorial neurovascular process extending beyond isolated large vessel vasospasm. This systematic review evaluated emerging neurovascular interventions for the prevention and management of cerebral vasospasm and delayed cerebral ischemia following aneurysmal subarachnoid hemorrhage. A comprehensive literature search was conducted across PubMed/MEDLINE, Scopus, and Web of Science databases for studies published between January 2020 and February 2026 in accordance with Preferred Reporting Items for Systematic Reviews and Meta-Analyses (PRISMA) 2020 guidelines. Nine studies met the eligibility criteria and were included in the qualitative synthesis. The included interventions encompassed endothelin receptor antagonists, localized nicardipine release implants, intra-arterial nimodipine infusion, microcirculatory targeted pharmacologic therapies, anti-inflammatory neuroprotective agents, and autonomic neuromodulatory approaches. Several interventions demonstrated reductions in angiographic vasospasm, delayed cerebral ischemia incidence, rescue therapy utilization, or cerebral infarction burden. However, consistent improvement in long-term neurological recovery remained variable across studies. Emerging evidence increasingly supports a broader pathophysiological model of delayed cerebral ischemia involving endothelial dysfunction, microcirculatory impairment, neuroinflammation, and autonomic dysregulation in addition to proximal arterial narrowing. Contemporary neurovascular interventions may therefore represent an important shift toward more targeted and mechanistically informed therapeutic strategies after aneurysmal subarachnoid hemorrhage, although further large-scale multicenter investigations remain necessary to establish definitive clinical benefit.

## Introduction and background

Aneurysmal subarachnoid hemorrhage (aSAH) remains one of the most devastating forms of cerebrovascular disease and is associated with substantial mortality and long-term neurological disability despite advances in neurosurgical and neurocritical care management [1,2]. Among survivors of the initial hemorrhagic event, delayed cerebral ischemia (DCI) continues to represent a major determinant of unfavorable outcome. Traditionally, cerebral vasospasm involving the large intracranial arteries was considered the principal mechanism underlying secondary ischemic injury after aSAH [3]. However, increasing evidence suggests that the pathophysiology of DCI is considerably more complex and likely involves an interplay of endothelial dysfunction, microcirculatory impairment, neuroinflammation, cortical spreading depolarizations, autonomic dysregulation, and disturbances in cerebral autoregulation.

Standard management strategies for vasospasm and DCI have historically relied on oral nimodipine, hemodynamic augmentation, and rescue endovascular interventions such as intra-arterial vasodilator infusion or balloon angioplasty. Although these approaches remain important components of clinical practice, their effects on long-term neurological recovery have been variable [4]. In parallel, a growing body of research has explored emerging neurovascular interventions aimed at modifying specific pathophysiological pathways implicated in secondary ischemic injury. These strategies include endothelin receptor antagonists, localized sustained release drug delivery systems, microcirculatory targeted pharmacologic agents, anti-inflammatory and neuroprotective therapies, autonomic neuromodulation techniques, and novel rescue endovascular pharmacologic approaches. Collectively, these interventions reflect an evolving therapeutic paradigm that extends beyond isolated large vessel vasospasm toward broader modulation of neurovascular dysfunction after aSAH [5].

Despite increasing therapeutic innovation, the available literature remains heterogeneous with respect to study design, intervention type, clinical endpoints, and mechanistic focus. Some interventions have demonstrated reductions in angiographic vasospasm or surrogate neurovascular markers without consistent improvement in functional outcomes, whereas others have shown encouraging signals in delayed ischemic injury reduction or neurological recovery despite limited sample sizes [6]. Furthermore, the relationship between vasospasm reduction and meaningful clinical benefit remains an area of ongoing investigation. As a result, careful synthesis of contemporary evidence is necessary to better characterize the current landscape of emerging neurovascular therapies and to identify areas where further clinical validation may be warranted [7].

The objective of this systematic review is to evaluate emerging neurovascular interventions for the prevention and management of cerebral vasospasm and delayed cerebral ischemia after aneurysmal subarachnoid hemorrhage. Specifically, this review aims to synthesize current evidence regarding targeted pharmacologic therapies, localized drug delivery systems, endovascular rescue approaches, and neuromodulatory interventions, with emphasis on their effects on vasospasm-related outcomes, delayed cerebral ischemia, cerebral infarction, and functional neurological recovery.

## Review

Materials and methods

Study Design and Reporting Framework

This systematic review was conducted to evaluate emerging neurovascular interventions for the prevention and management of cerebral vasospasm and delayed cerebral ischemia following aneurysmal subarachnoid hemorrhage. The review methodology was developed in accordance with the Preferred Reporting Items for Systematic Reviews and Meta-Analyses (PRISMA) 2020 guidelines to ensure transparent study identification, screening, eligibility assessment, and evidence synthesis [8]. The research question was structured using the Population, Intervention, Comparator, and Outcomes (PICO) framework [9]. The population included adult patients with aneurysmal subarachnoid hemorrhage. The interventions of interest included emerging pharmacologic, endovascular, localized drug delivery, and neuromodulatory neurovascular therapies targeting cerebral vasospasm or delayed cerebral ischemia. Comparators included placebo, sham stimulation, standard medical management, or conventional neurocritical care protocols. The primary outcomes of interest included cerebral vasospasm, delayed cerebral ischemia, cerebral infarction, rescue therapy utilization, and neurological functional outcomes assessed using validated clinical scales.

Literature Search Strategy

A comprehensive literature search was conducted using PubMed/MEDLINE, Scopus, and Web of Science databases for studies published between January 2020 and February 2026. Combinations of Medical Subject Headings (MeSH) terms, free-text keywords, and Boolean operators ("AND" and "OR") were applied to optimize search sensitivity and specificity for studies related to aneurysmal subarachnoid hemorrhage, cerebral vasospasm, delayed cerebral ischemia, neurovascular interventions, neuromodulation, and targeted pharmacologic therapies. Representative database search strategies and keyword combinations are summarized in Table 1. Reference lists of relevant studies and review articles were also manually screened to identify additional eligible studies.

**Table 1:** Representative database search strategy used for literature identification

Database	Search terms and keywords used	Search strategy / combination approach
PubMed/MEDLINE	"aneurysmal subarachnoid hemorrhage", "aSAH", "cerebral vasospasm", "delayed cerebral ischemia", "clazosentan", "nicardipine", "cilostazol", "intra arterial nimodipine", "stellate ganglion block", "trigeminal nerve stimulation", "endovascular therapy", "neuromodulation"	Multiple combinations of MeSH terms and free-text keywords were applied using Boolean operators ("AND" and "OR") to optimize search sensitivity and specificity
Scopus	"aneurysmal subarachnoid hemorrhage". "cerebral vasospasm", "delayed cerebral ischemia", "clazosentan", "nicardipine", "cilostazol", "endovascular therapy", "neuromodulation", "targeted pharmacologic therapy"	Different keyword combinations and Boolean search strategies were used across title, abstract, and indexed keyword fields
Web of Science	"aneurysmal subarachnoid hemorrhage", "aSAH", "delayed cerebral ischemia", "vasospasm", "neurovascular intervention", "trigeminal nerve stimulation", "stellate ganglion block"	Search combinations incorporating neurovascular intervention concepts and delayed cerebral ischemia terminology were applied using Boolean operators and topic field searching

Eligibility Criteria

Eligibility criteria were predefined based on study design, population, intervention type, outcomes, and publication characteristics. Studies evaluating targeted neurovascular interventions for the prevention or management of cerebral vasospasm or delayed cerebral ischemia after aneurysmal subarachnoid hemorrhage were considered eligible. Detailed inclusion and exclusion criteria are summarized in Table 2.

**Table 2:** Inclusion and exclusion criteria used for study selection aSAH - aneurysmal subarachnoid hemorrhage; DCI - delayed cerebral ischemia

Category	Inclusion criteria	Exclusion criteria
Population	Adult patients with aneurysmal subarachnoid hemorrhage	Pediatric populations or non-aSAH populations
Study design	Randomized controlled trials, prospective clinical trials, and mechanistically focused interventional studies	Non-interventional observational studies, case reports, conference abstracts, editorials, and narrative reviews
Intervention	Pharmacologic therapies, localized neurovascular drug delivery systems, endovascular rescue therapies, and neuromodulatory interventions targeting vasospasm or delayed cerebral ischemia	Studies unrelated to neurovascular intervention or vasospasm/DCI management
Outcomes	Studies reporting angiographic vasospasm, delayed cerebral ischemia, cerebral infarction, rescue therapy utilization, or functional neurological outcomes	Studies lacking clinically relevant neurovascular outcome data
Publication characteristics	English-language studies published between January 2020 and February 2026	Non-English studies or studies published outside the predefined study period
Mechanistic focus	Studies specifically addressing neurovascular complications after aneurysmal subarachnoid hemorrhage	Prognostic biomarker studies, imaging prediction model studies, animal studies, and surgical technique comparisons unrelated to vasospasm management

Study Selection and Data Extraction

All identified records underwent title and abstract screening followed by full-text eligibility assessment. Duplicate records were removed prior to screening. Studies meeting the predefined eligibility criteria were included in the qualitative synthesis. Data extraction was performed systematically using a standardized evidence extraction framework designed for this review. Extracted variables included study design, patient population, intervention category, intervention characteristics, comparator group, vasospasm and delayed cerebral ischemia outcomes, functional neurological outcomes, and principal study conclusions. Particular attention was given to mechanistic themes, including endothelial modulation, microcirculatory dysfunction, autonomic neuromodulation, inflammatory signaling, and targeted neurovascular rescue strategies.

Risk of Bias Assessment

Risk of bias assessment was conducted using the revised Cochrane Risk of Bias 2 (RoB 2) tool [10] for randomized controlled trials. For secondary analyses derived from randomized trial populations or studies with partially non-randomized methodological characteristics, the Risk Of Bias In Non-randomized Studies of Interventions (ROBINS-I) tool [11] was applied where appropriate. Domains assessed included the randomization process, deviations from intended interventions, missing outcome data, outcome measurement, and selective reporting. Overall judgments were categorized as low risk, some concerns, or moderate risk of bias according to established methodological guidance. Given the heterogeneity of interventions and outcome reporting, a qualitative narrative synthesis approach was considered most appropriate for evidence integration.

Results

Study Selection Process

**Figure 1:**
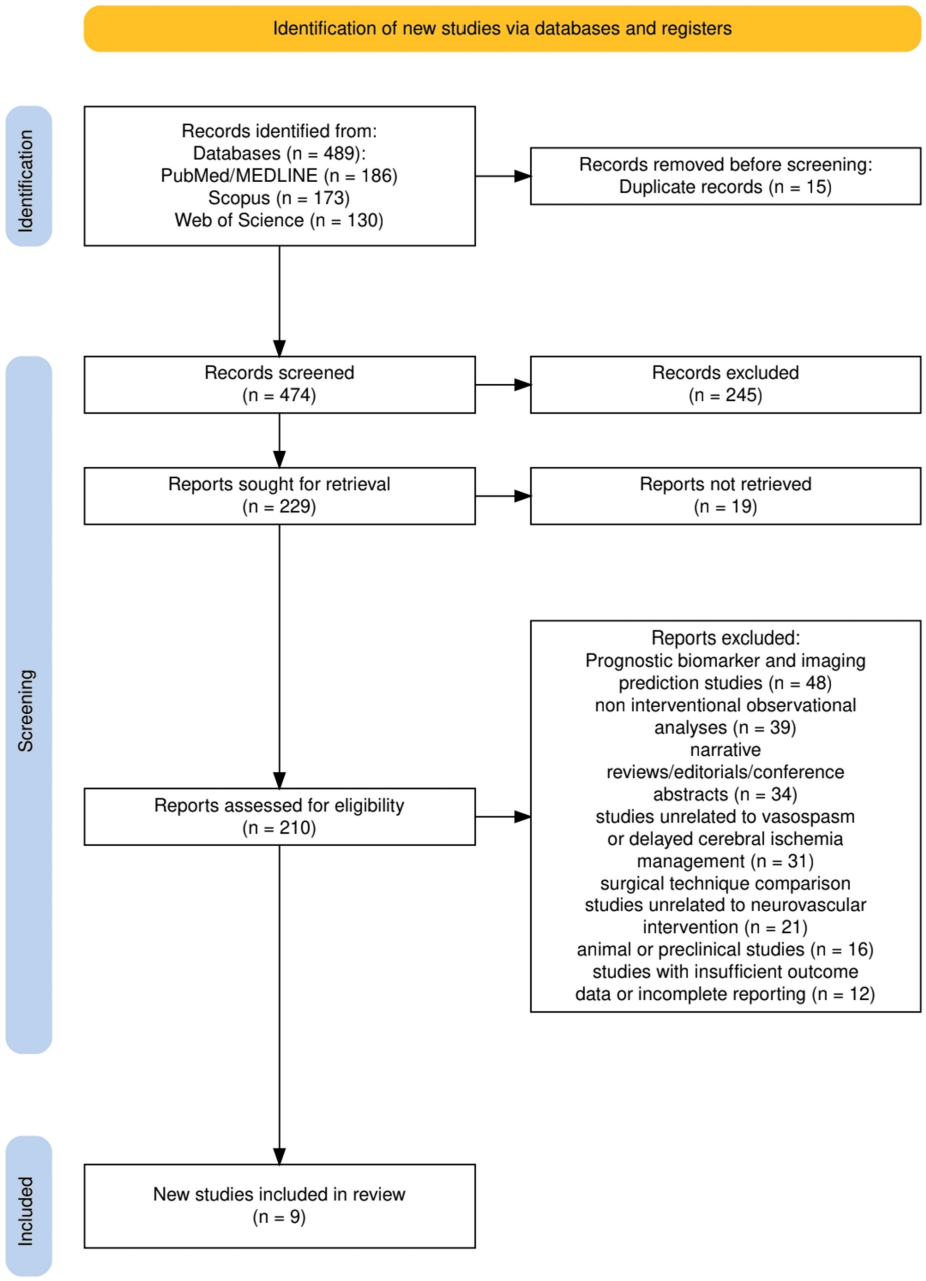
The PRISMA flow diagram represents the study selection process PRISMA - Preferred Reporting Items for Systematic Reviews and Meta-Analyses

The study selection process is summarized in Figure 1. A total of 489 records were initially identified through database searching, including PubMed/MEDLINE, Scopus, and Web of Science. After removal of duplicate records, 474 studies underwent title and abstract screening, of which 245 were excluded based on predefined eligibility criteria. Full-text assessment was performed for 210 reports after exclusion of unretrieved articles. The most common reasons for exclusion included prognostic biomarker or imaging prediction studies, non- interventional observational analyses, narrative reviews and editorials, studies unrelated to vasospasm or delayed cerebral ischemia management, surgical technique comparison studies unrelated to neurovascular intervention, animal or preclinical investigations, and studies with insufficient outcome reporting. Ultimately, nine studies met the eligibility criteria and were included in the final qualitative synthesis.

Characteristics of the Selected Studies

The included studies consisted predominantly of randomized controlled trials evaluating emerging neurovascular interventions for the prevention or management of cerebral vasospasm and delayed cerebral ischemia after aneurysmal subarachnoid hemorrhage. The interventions investigated included endothelin receptor antagonists, localized pharmacologic delivery systems, intra-arterial rescue therapies, anti-inflammatory and microcirculatory targeted agents, as well as autonomic and trigeminal neuromodulatory approaches. Study populations largely involved adult patients with secured aneurysmal subarachnoid hemorrhage following surgical clipping or endovascular coiling, with several studies focusing on patients at increased risk of vasospasm based on hemorrhage severity or angiographic findings. The primary outcomes commonly included angiographic vasospasm, delayed cerebral ischemia, cerebral infarction, rescue therapy utilization, and neurological functional recovery assessed using validated outcome scales. While some studies demonstrated clinically meaningful reductions in vasospasm-related complications, others primarily reported mechanistic or physiologic effects without definitive long-term functional benefit. A detailed summary of study characteristics is presented in Table 3.

**Table 3:** Characteristics of randomized controlled trials evaluating emerging neurovascular interventions for prevention of cerebral vasospasm and delayed cerebral ischemia after aneurysmal subarachnoid hemorrhage aSAH - aneurysmal subarachnoid hemorrhage; DCI - delayed cerebral ischemia; IV - intravenous; GOSE - Glasgow Outcome Scale-Extended; mRS - modified Rankin scale; WFNS - World Federation of Neurosurgical Societies; iCCT - intracerebral circulation time; IANI - intra-arterial nimodipine infusion; CSF - cerebrospinal fluid; EET - epoxyeicosatrienoic acid; DHET - dihydroxyeicosatrienoic acid; IA - intra-arterial; SGB - stellate ganglion block; TCD - transcranial Doppler

Study	Design	Population	Intervention category	Intervention	Comparator	Vasospasm/DCI outcomes	Functional outcomes	Key conclusion
Mayer et al., 2024 [12]	Prospective multicenter double-blind phase 3 randomized trial	409 aSAH patients after clipping or coiling with thick/diffuse clot burden	Endothelin receptor antagonist therapy	IV clazosentan + standard care	Placebo + standard care	No significant reduction in DCI-related clinical deterioration; reduced rescue therapy utilization; trend toward fewer cerebral infarcts	No significant improvement in GOSE or mRS outcomes	Clazosentan reduced rescue therapy requirement but did not significantly improve DCI-related clinical deterioration or functional outcomes
Endo et al., 2022 [13]	Double-blind placebo-controlled phase 3 randomized trials	440 Japanese aSAH patients after clipping or coiling	Endothelin receptor antagonist therapy	IV clazosentan	Placebo	Significant reduction in vasospasm-related morbidity/mortality, delayed ischemic deficits, and cerebral infarction	Improved pooled mRS outcomes after study pooling	Clazosentan significantly reduced vasospasm-related morbidity and mortality after aSAH with acceptable safety
Wessels et al., 2024 [14]	Single-masked randomized clinical trial	41 aSAH patients with WFNS grade 3–4 undergoing microsurgical aneurysm repair	Localized neurovascular pharmacologic delivery	Intraoperative nicardipine release implants + standard care	Standard care alone	Significant reduction in moderate-to-severe angiographic vasospasm and rescue therapy requirement; lower infarction trend	Higher favorable outcome rate at 52 weeks, though not statistically significant	Localized nicardipine implants safely reduced angiographic vasospasm and rescue intervention need after aSAH
Naraoka et al., 2025 [15]	Analysis of patients from two randomized controlled trials	256 aSAH patients	Microcirculatory-targeted pharmacologic therapy	Cilostazol	Pitavastatin and control groups	Reduced DCI incidence, improved intracerebral circulation time (iCCT), attenuated microcirculatory dysfunction	Cilostazol treatment independently predicted favorable outcomes	Cilostazol may alleviate DCI and improve outcomes by improving cerebral microcirculatory dysfunction after aSAH
Yindeedej et al., 2022 [16]	Prospective randomized controlled trial	68 aSAH patients with angiographic cerebral vasospasm	Endovascular pharmacologic rescue therapy	Adjunct intra-arterial nimodipine infusion (IANI)	Standard medical therapy alone	Significant early neurologic and motor improvement after vasospasm treatment	Improved motor recovery at discharge without significant complications	IANI may be an effective and safe rescue treatment for cerebral vasospasm after aSAH
Martini et al., 2022 [17]	Double-blind randomized placebo-controlled phase Ib trial	19 aSAH patients	Neurovascular anti-inflammatory pharmacologic therapy	GSK2256294 (soluble epoxide hydrolase inhibitor)	Placebo	Increased serum EET/DHET ratio; trend toward reduced CSF inflammatory cytokines; exploratory DCI assessment	No major clinical outcome differences demonstrated; therapy was well tolerated	sEH inhibition was safe and demonstrated biologic activity supporting future investigation for DCI prevention
García-Pastor et al., 2022 [18]	Prospective randomized double-blind placebo-controlled trial	48 aSAH patients with Fisher grade 3–4	Neuroprotective/ anti-inflammatory pharmacologic therapy	Dapsone 100 mg daily + standard care	Placebo + standard care	Significant reduction in DCI incidence, irreversible DCI, and brain infarction; lower requirement for IA nimodipine rescue therapy	Improved mRS outcomes at discharge and three months	Dapsone demonstrated potential neuroprotective effects with reduced DCI and improved functional outcomes after aSAH
Wu et al., 2023 [19]	Pilot randomized controlled trial	aSAH patients undergoing surgical treatment	Autonomic neuromodulation / sympathetic blockade	Early stellate ganglion block (SGB)	Standard management without SGB	Lower symptomatic vasospasm incidence, reduced TCD-defined vasospasm, lower cerebral blood flow velocity, fewer new cerebral infarctions	Trend toward improved prognosis without increased adverse events or mortality	Early SGB showed potential to reduce vasospasm burden and cerebral infarction after aSAH
Rigoard et al., 2023 [20]	Double-blind randomized pilot trial	60 aSAH patients (WFNS 1–4)	Neuromodulation / targeted neurovascular intervention	Transcutaneous trigeminal nerve stimulation (TNS)	Sham stimulation	No significant reduction in vasospasm-related cerebral infarction or DCI	No significant difference at three-month follow-up	TNS did not significantly reduce vasospasm-related infarction, though the concept warrants further investigation

Quality Assessment

Risk of bias assessment demonstrated generally acceptable methodological quality across the included studies, although several limitations were identified. Most randomized controlled trials showed low risk or some concerns across key RoB 2 domains, particularly regarding randomization procedures, outcome measurement, and deviations from intended interventions. Larger multicenter phase 3 trials generally exhibited stronger methodological rigor and more standardized outcome assessment, whereas several pilot and exploratory studies were limited by small sample sizes, incomplete blinding, and reduced statistical power for long-term functional outcomes. In addition, heterogeneity in delayed cerebral ischemia definitions, follow-up durations, and endpoint selection contributed to variability in methodological robustness. The secondary analysis study included in this review demonstrated a moderate risk of bias because the evaluated mechanistic associations were not derived from a primary randomized allocation designed specifically for the review question. Overall, although several studies demonstrated promising biologic and neurovascular effects, caution remains necessary when interpreting clinical efficacy outcomes due to methodological heterogeneity and limited external validity in certain cohorts. A detailed summary of the risk of bias assessment is presented in Table 4.

**Table 4:** Risk of bias assessment of randomized and mechanistic intervention studies evaluating neurovascular therapies after aneurysmal subarachnoid hemorrhage. RoB 2 - Cochrane Risk of Bias 2 tool; ROBINS-I - Risk Of Bias In Non-randomized Studies of Interventions; aSAH - aneurysmal subarachnoid hemorrhage; DCI - delayed cerebral ischemia

Study	Suitable tool	Randomization process	Deviations from intended intervention	Missing outcome data	Outcome measurement	Selective reporting	Overall risk of bias
Mayer et al., 2024 [12]	RoB 2	Low	Low	Low	Low	Low	Low risk
Endo et al., 2022 [13]	RoB 2	Low	Low	Low	Low	Some concerns	Some concerns
Wessels et al., 2024 [14]	RoB 2	Some concerns	Some concerns	Some concerns	Low	Some concerns	Some concerns
Naraoka et al., 2025 [15]	ROBINS-I	Moderate	Moderate	Moderate	Some concerns	Moderate	Moderate risk
Yindeedej et al., 2022 [16]	RoB 2	Some concerns	Some concerns	Some concerns	Some concerns	Some concerns	Some concerns
Martini et al., 2022 [17]	RoB 2	Low	Low	Some concerns	Low	Some concerns	Some concerns
García-Pastor et al., 2022 [18]	RoB 2	Some concerns	Low	Some concerns	Some concerns	Some concerns	Some concerns
Wu et al., 2023 [19]	RoB 2	Some concerns	Some concerns	Some concerns	Some concerns	Some concerns	Some concerns
Rigoard et al., 2023 [20]	RoB 2	Low	Low	Some concerns	Low	Some concerns	Some concerns

Discussion

Principal Findings

Contemporary neurovascular interventions for aneurysmal subarachnoid hemorrhage increasingly reflect a transition from isolated vasospasm suppression toward broader modulation of delayed cerebral ischemic injury. Across the included studies, reduction in angiographic vasospasm burden, delayed cerebral ischemia incidence, rescue therapy utilization, or surrogate neurovascular markers was observed more consistently than robust improvement in long-term neurological recovery. Large randomized trials by Mayer et al. [12] and Endo et al. [13] demonstrated that endothelin receptor antagonism with clazosentan could reduce vasospasm-related morbidity and rescue intervention requirements, although the translation of these effects into sustained functional benefit remained variable. Similarly, Wessels et al. [14] reported meaningful reductions in angiographic vasospasm and rescue therapy use with localized nicardipine implants, while studies evaluating intra-arterial nimodipine, autonomic modulation, and anti-inflammatory pharmacologic strategies suggested physiologic and clinical signals of benefit despite relatively limited sample sizes. Collectively, these findings suggest that emerging interventions may favorably influence selected neurovascular and ischemic endpoints after aneurysmal subarachnoid hemorrhage, although consistent improvement in global neurological recovery remains incompletely established.

Evolving Understanding of Delayed Cerebral Ischemia

The findings synthesized in this review also support the evolving concept that delayed cerebral ischemia represents a multifactorial neurovascular syndrome rather than solely a consequence of proximal large vessel narrowing. Increasing attention has been directed toward mechanisms involving microcirculatory dysfunction, endothelial injury, neuroinflammation, autonomic dysregulation, and impaired cerebrovascular coupling. Naraoka et al. [15] demonstrated that cilostazol was associated with improved intracerebral circulation time and attenuation of microcirculatory dysfunction, supporting the notion that distal perfusion abnormalities may substantially contribute to ischemic injury after aneurysmal subarachnoid hemorrhage. Similarly, Martini et al. [17] explored soluble epoxide hydrolase inhibition as a strategy targeting inflammatory and endothelial signaling pathways involved in cerebral blood flow regulation, whereas García-Pastor et al. [18] reported reduced delayed cerebral ischemia and infarction rates with dapsone, suggesting a potential role for anti-inflammatory neuroprotection. In parallel, neuromodulatory interventions investigated by Wu et al. [19] and Rigoard et al. [20] highlighted the potential influence of autonomic pathways and trigeminal vascular regulation on cerebral hemodynamics and vasospasm physiology. Taken together, these studies reinforce a broader pathophysiological framework in which delayed cerebral ischemia emerges from interacting disturbances across vascular, inflammatory, microcirculatory, and neuroregulatory domains rather than from angiographic vasospasm alone.

Angiographic Response and Functional Recovery

An important finding across the contemporary literature is the imperfect relationship between vasospasm reduction and meaningful neurological recovery. Trials by Mayer et al. [12], Endo et al. [13], and Wessels et al. [14] demonstrate this tension particularly well. Although clazosentan and localized nicardipine delivery showed favorable effects on vasospasm-related endpoints, rescue therapy use, or vasospasm-related morbidity, the translation into consistent long-term functional improvement was less uniform. This discordance suggests that angiographic vasospasm, while clinically important, may not fully capture the biological complexity of delayed cerebral ischemia or the determinants of recovery after aneurysmal subarachnoid hemorrhage. Early brain injury, microvascular dysfunction, inflammatory activation, cortical spreading depolarizations, impaired autoregulation, systemic complications, and delayed secondary injury cascades may continue to influence outcome even when proximal vessel narrowing is reduced. Therefore, successful modulation of angiographic vasospasm alone may be insufficient to substantially alter long-term neurological recovery.

Targeted and Localized Neurovascular Therapies

The included studies also illustrate an emerging movement from broad systemic therapy toward more precise neurovascular modulation. Wessels et al. [14] evaluated localized nicardipine release implants placed around vulnerable basal vessels, representing a targeted drug delivery approach designed to concentrate therapy at the site of greatest vasospasm risk while potentially limiting systemic exposure. Yindeedej et al. [16] extended this precision-oriented concept into rescue therapy by examining intra-arterial nimodipine infusion for established angiographic vasospasm, allowing direct pharmacologic treatment of affected cerebral vessels. Similarly, Wu et al. [19] investigated stellate ganglion block as a method of sympathetic modulation, while Rigoard et al. [20] explored trigeminal nerve stimulation as a neuromodulatory strategy intended to influence cerebrovascular tone through autonomic pathways. Although the strength of evidence remains variable, these studies collectively suggest that future management of vasospasm and delayed cerebral ischemia may increasingly rely on targeted interventions tailored to vascular territory, timing, mechanism, and patient risk profile.

Rescue Therapy and Escalation Strategies

Rescue therapy remains an important component of vasospasm management because preventive neurovascular interventions have not consistently eliminated delayed ischemic complications after aneurysmal subarachnoid hemorrhage. This is reflected in the clazosentan trials, where Mayer et al. [12] reported reduced rescue therapy utilization despite no significant improvement in the primary delayed cerebral ischemia endpoint, while Endo et al. [13] demonstrated reductions in vasospasm-related morbidity with endothelin receptor antagonism. These findings suggest that effective prevention may reduce the need for escalation, but does not fully replace the clinical role of rescue intervention. In this context, Yindeedej et al. [16] provide particularly relevant evidence by evaluating intra-arterial nimodipine infusion for established angiographic vasospasm, supporting the continued importance of direct endovascular pharmacologic therapy when vasospasm becomes clinically significant or refractory to standard management. A balanced therapeutic paradigm may therefore require both upstream prevention and downstream salvage strategies, with escalation guided by clinical deterioration, vascular imaging, perfusion assessment, and institutional expertise.

Methodological and Evidence Limitations

The interpretation of this evidence base requires caution because many included studies were limited by small pilot populations, heterogeneous endpoints, variable definitions of delayed cerebral ischemia, and inconsistent follow-up durations. Several trials emphasized angiographic vasospasm, cerebral blood flow velocity, inflammatory biomarkers, or microcirculatory measures, whereas others prioritized clinical deterioration, infarction, rescue therapy use, or functional outcome scores. This variability complicates direct comparison across interventions and may partly explain why biologic or physiologic signals did not consistently translate into definitive clinical efficacy. In addition, several studies were underpowered to detect meaningful differences in long-term functional recovery, particularly when favorable outcomes were assessed at different time points or using different scales. External validity also remains limited in studies restricted to selected surgical populations, single-center cohorts, or specific regional practice settings. Finally, standardizing neurocritical care protocols across trials is inherently difficult because background management, rescue thresholds, imaging surveillance, hemodynamic strategies, and institutional endovascular capabilities may substantially influence observed outcomes.

Future Directions

Future research may increasingly move toward multimodal neurovascular management strategies that integrate pharmacologic, endovascular, and neuromodulatory approaches rather than relying on isolated intervention pathways. The evolving understanding of delayed cerebral ischemia as a heterogeneous neurovascular process suggests that individualized risk stratification and biomarker-guided intervention may represent important areas for further investigation. In particular, incorporation of microcirculatory assessment, endothelial and inflammatory biomarkers, advanced perfusion imaging, and physiologic neuromonitoring may help clarify which patients are most likely to benefit from targeted therapies. Combination approaches involving pharmacologic modulation alongside autonomic or endovascular interventions also warrant further investigation, especially in patients with refractory or high-risk vasospasm phenotypes. Nevertheless, the optimal timing, patient selection criteria, and mechanistic interactions among these therapies remain incompletely understood. Future studies should therefore prioritize standardized delayed cerebral ischemia definitions, clinically meaningful functional outcomes, and larger multicenter designs capable of evaluating integrated precision neurocritical care strategies.

## Conclusions

Current evidence suggests that emerging neurovascular interventions may reduce vasospasm burden and certain ischemic complications after aneurysmal subarachnoid hemorrhage, although consistent improvement in long-term functional recovery remains uncertain. Contemporary research increasingly supports a broader neurovascular model of delayed cerebral ischemia involving microcirculatory dysfunction, neuroinflammation, endothelial injury, and autonomic dysregulation rather than isolated angiographic vasospasm alone. The included studies collectively reflect an evolving therapeutic landscape characterized by targeted pharmacologic modulation, localized drug delivery systems, endovascular rescue strategies, and neuromodulatory interventions. While several therapies demonstrated promising physiologic or mechanistic effects, further high-quality multicenter investigations are needed to determine whether these advances can reliably translate into sustained neurological recovery and improved patient-centered outcomes after aneurysmal subarachnoid hemorrhage.
